# Identification of Molecular Basis for Objective Discrimination of Breast Cancer Cells (MCF-7) from Normal Human Mammary Epithelial Cells by Raman Microspectroscopy and Multivariate Curve Resolution Analysis

**DOI:** 10.3390/ijms22020800

**Published:** 2021-01-14

**Authors:** Keita Iwasaki, Asuka Araki, C Murali Krishna, Riruke Maruyama, Tatsuyuki Yamamoto, Hemanth Noothalapati

**Affiliations:** 1 The United Graduate School of Agricultural Sciences, Tottori University, Tottori 680-8550, Japan; d17a3003@matsu.shimane-u.ac.jp; 2 Department of Organ Pathology, Faculty of Medicine, Shimane University, Izumo 693-8501, Japan; asuka@med.shimane-u.ac.jp (A.A.); hm5995@med.shimane-u.ac.jp (R.M.); 3 Advanced Centre for Treatment, Research and Education in Cancer, Tata Memorial Centre, Navi Mumbai 410-210, India; pittu1043@gmail.com; 4 Faculty of Life and Environmental Science, Shimane University, Matsue 690-8504, Japan; 5 Raman Project Center for Medical and Biological Applications, Shimane University, Matsue 690-8504, Japan; 6 Research Administration Office, Shimane University, Matsue 690-8504, Japan

**Keywords:** Raman spectroscopy, breast cancer, linoleic acid, lipid metabolism, PUFA, cancer diagnosis, MCR-ALS, chemometrics, cpectral marker, cytodiagnosis

## Abstract

Raman spectroscopy (RS), a non-invasive and label-free method, has been suggested to improve accuracy of cytological and even histopathological diagnosis. To our knowledge, this novel technique tends to be employed without concrete knowledge of molecular changes in cells. Therefore, identification of Raman spectral markers for objective diagnosis is necessary for universal adoption of RS. As a model study, we investigated human mammary epithelial cells (HMEpC) and breast cancer cells (MCF-7) by RS and employed various multivariate analyses (MA) including principal components analysis (PCA), linear discriminant analysis (LDA), and support vector machine (SVM) to estimate diagnostic accuracy. Furthermore, to elucidate the underlying molecular changes in cancer cells, we utilized multivariate curve resolution analysis–alternating least squares (MCR-ALS) with non-negative constraints to extract physically meaningful spectra from complex cellular data. Unsupervised PCA and supervised MA, such as LDA and SVM, classified HMEpC and MCF-7 fairly well with high accuracy but without revealing molecular basis. Employing MCR-ALS analysis we identified five pure biomolecular spectra comprising DNA, proteins and three independent unsaturated lipid components. Relative abundance of lipid 1 seems to be strictly regulated between the two groups of cells and could be the basis for excellent discrimination by chemometrics-assisted RS. It was unambiguously assigned to linoleate rich glyceride and therefore serves as a Raman spectral marker for reliable diagnosis. This study successfully identified Raman spectral markers and demonstrated the potential of RS to become an excellent cytodiagnostic tool that can both accurately and objectively discriminates breast cancer from normal cells.

## 1. Introduction

Despite advances in prognosis and treatment, cancer incidence and mortality are rapidly increasing around the world. According to the estimates by the International Agency for Research on Cancer in 2018, there were about 18.1 million new cases and 9.6 million cancer deaths. Among women, breast cancer with 2.1 million cases and over 0.6 million deaths tops the chart [1]. Breast cancer can be diagnosed through multiple tests including an X-ray mammogram, ultrasound imaging, magnetic resonance imaging, fine needle aspiration cytology, and tissue biopsy etc. Presently, histopathology remains to be a gold standard in breast cancer diagnosis and treatment. However, identification of molecular signatures using this invasive procedure is expensive, involves tedious sample preparation, is time consuming and sometimes leads to ambiguous results due to human interpretations. Thus, it has severe limitations especially during surgeries. Therefore, it is necessary to develop alternative methods that are low- or non-invasive and economical while achieving rapid diagnosis with high accuracy.

Raman spectroscopy (RS), a powerful vibrational spectroscopic technique based on inelastic scattering of light, has been proposed to be a good alternative to overcome such difficulties. Advantages of RS are manifold: (1) non-invasive, i.e., suitability to in vivo applications, (2) no need for staining or genetic manipulation, (3) high sensitivity and specificity due to rich molecular information. Indeed, RS has been gaining much attention and has been successfully applied in disease prognosis and diagnosis [2,3,4], discriminate cells and tissues [5,6], image living cells in a label-free manner [7,8] and probe metabolic pathways [9,10]. However, there are limitations to RS as well. First, traditional raster scanning methods employed in RS are extremely slow procedures, especially when considering the size of the tissues examined during histopathology. To solve this problem, researchers have proposed various methods, such as hand-held Raman probes for guided biopsy [11] and autofluorescence combined with selective Raman sampling, [12] etc. Second, since RS measures molecular vibrations, different molecules containing similar chemical bonds show similar frequencies and, in most cases, it is not appropriate to simply use a single band for spectral interpretation. To make matters worse, Raman hyperspectral imaging results in a large volume of data with thousands of Raman spectra to handle. Therefore, we need to employ multivariate analyses (MA) for meaningful interpretation. To this end, a variety of multivariate analytical methods have been developed. Some of the most popular unsupervised multivariate classification methods applied to Raman spectroscopic data include singular value decomposition (SVD), principal components analysis (PCA), and cluster analysis, etc., which are suitable for exploratory analysis. On the other hand, if a priori information about the samples is available, supervised methods such as linear discriminant analysis (LDA), neural networks, and support vector machine (SVM) etc., are well suited to model the given Raman hyperspectral data and apply it to predict unknown samples.

It is surprising to note that some of the early studies demonstrating the potential application of RS to cancers was done by Manfait and co-workers as early as 1982 [13,14]. This was followed by several studies especially focusing on RS-based breast cancer diagnosis in the early nineties [15,16]. Due to technological advancements and the development of chemometrics in the last two decades, the volume of RS-based literature has kept growing rapidly. To put things into perspective, a simple ‘Topic’ search with a keyword ‘Raman AND Cancer’ in the Web of Science database returned ~5000 documents. Even though application of RS has been proven to be successful under laboratory situations, it is important to understand that these MA results are subjective to many factors, including design of experiment and analysis, data pre-processing and overall quality of data. Therefore, experience of the person, instrument performance and acquisition parameters also play a crucial role. Owing to limitations in standardizing the whole procedure, universal adoption of RS in clinics has still not been achieved. Another major drawback is that none of the above-mentioned MA procedures discriminate/classify/predict based on inherent chemical information but strictly treat Raman spectroscopic data only mathematically. Therefore, to overcome these limitations, we employed an alternative approach called multivariate curve resolution-alternating least squares (MCR-ALS) in which pure chemical components and their abundances are extracted from Raman hyperspectral data to establish a molecular basis for reliable diagnosis. In this study, we identified for the first time that linoleate rich triglycerides serve as the marker for objective discrimination of MCF-7 and HMEpC cells in 632.8 nm excited chemometrics assisted Raman microspectroscopy.

## 2. Results

### 2.1. Univariate Analysis of Normal and MCF-7 Cells Gives Little Information for Objective Discrimination

**Figure 1:**
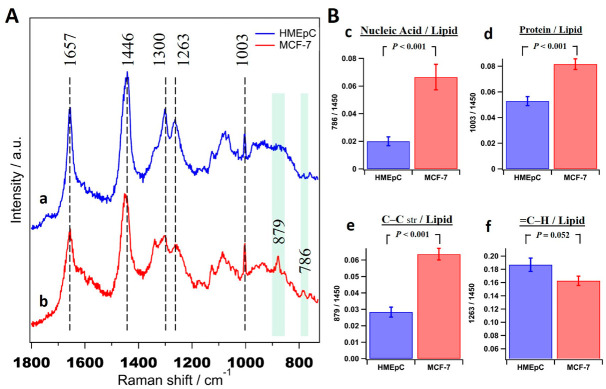
Comparison of average Raman spectra of MCF-7 and HMEpC. (**A**) Averaged Raman spectra (± S.D.) obtained from 30 cells of (**a**) HMEpC and (**b**) MCF-7, respectively. The consistent band positions were shown with broken lines and significant differences were highlighted by shaded bars. (**B**) Biomolecular ratios of (**c**) nucleic acid/lipid, (**d**) protein/lipid, (**e**) C–C str/lipid (chain lengths) and (**f**) =C–H/lipid (unsaturation).

Average Raman spectra of 30 cells each of normal human mammary epithelial cells (HMEpC) and breast cancer cells (MCF-7) are presented in Figure 1A. Some of the prominent bands observed in both spectra such as 1657 cm^−1^ (amide Ⅰ/-C=C- str), 1446 cm^−1^ (CH2/CH3), 1300 cm^−1^ (CH2 twisting), 1263 cm^−1^ (=C-H), and 1003 cm^−1^ (Phenyl alanine) indicate the contribution of proteins and lipids. Raman bands at 879 cm^−1^ and 786 cm^−1^ observed in cancer cells can be assigned to C-C stretch (protein, amino acid hydroxyproline and lipids) and O-P-O symmetric stretch (nucleic acids), respectively [17,18]. Since simple comparison only suggests general variation in proteins and nucleic acids, we integrated intensities of important Raman bands, calculated ratios of various biomacromolecules for each cell, and their averages along with standard deviations (S.D.) were used to identify markers for discrimination as shown in Figure 1B. Some ratios such as nucleic acid/lipid (Figure 1B(c)), protein/lipid (Figure 1B(d)) and C-C str/lipid (Figure 1B(e)) show significant differences between normal and cancer cells. However, it is important to note that these are calculated by univariate approach (using one representative band/species) and it is impossible to avoid band overlaps from other components in the same region. For example, band around ~1440 cm^−1^ has been traditionally used as a lipid marker but it originally represents CH2 and CH3 vibrations, which inevitably contains contributions from most other biomolecules. Therefore, instead of single band analysis, there is a need for multivariate methods that consider the whole spectrum for reliable diagnosis.

### 2.2. Application of Multivariate Statistical Methods to Discriminate Cancer Cells

To develop Raman spectroscopy as a diagnostic tool, it is imperative to detect subtle biochemical changes in disease conditions by employing multivariate statistics. In order to identify spectral differences and discriminate normal/cancer cells, we averaged only those spectra obtained from five different points in a cell and retained Raman spectrum representative of each individual cell for further analysis (60 spectra in total).

#### 2.2.1. Principal Components Analysis

**Figure 2:**
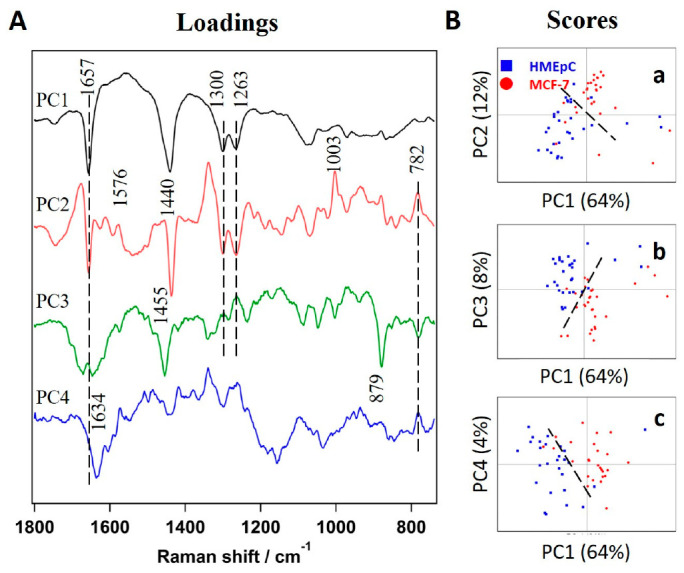
Results of principal components analysis (PCA) analysis. (**A**) First 4 principal components (PC) loadings, PC1 (64 %), PC2 (17%), PC3 (8%), and PC4 (4%). Broken lines show same band positions regardless of positive or negative tendency. (**B**) Scores plots of (**a**) PC2, (**b**) PC3 and (**c**) PC4 vs. PC1, respectively. Broken lines are drawn as visual guides to discriminate HMEpC and MCF-7.

PCA essentially reduces the dimensionality of hyperspectral data to a few principal components (PC) without losing much information. Indeed, it is one of the oldest and widely used multivariate methods in data analysis and has previously been applied to Raman spectroscopic data from cancer cells and tissues. Results of PCA showed a good degree of classification of the two groups of cells. PCA identified 7 PCs. The first four components that contribute 88% are presented in Figure 2. PC scores indicate PC1 to be the main contributor (64%) as it essentially can classify efficiently when taken with any of the next three PCs (Figure 2B). A closer look into loadings (Figure 2A) reveals that PC1 spectrum is dominated by bands of lipid origin such as 1657 cm^−1^, 1440 cm^−1^, 1300 cm^−1^, and 1263 cm^−1^. In addition to these bands in PC1, PC2 showed markers of protein (1003 cm^−1^) and nucleic acids (782 cm^−1^ and 1576 cm^−1^). Nucleic acid marker band at 1576 cm^−1^, which was not clearly observed in the average spectra, can be seen in PCA. Although PC loadings may provide molecular information to some degree, it is important to note that all of them show both positive and negative features. Moreover, most of the bands are mixed and are observed in multiple loadings, making it wrong to interpret the data in a physically meaningful way.

#### 2.2.2. Linear Discriminant Analysis

**Figure 3:**
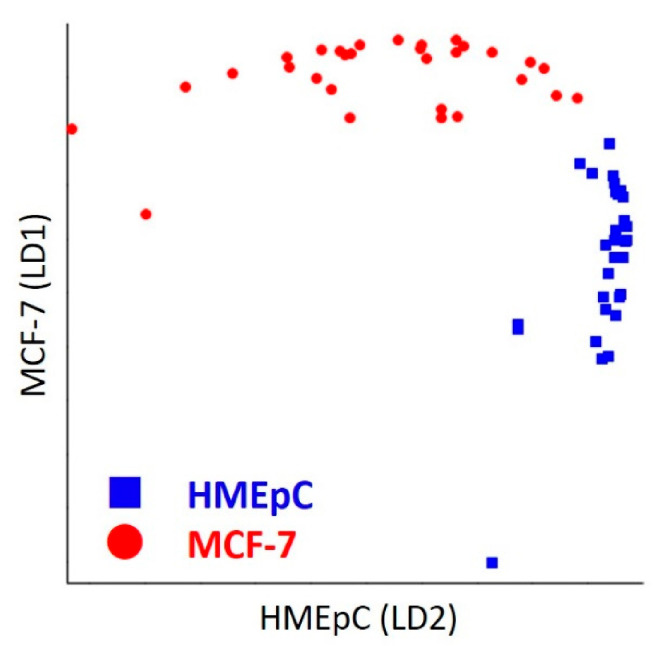
LDA Discrimination Plot. Linear discrimination factors of normal HMEpC and breast cancer MCF-7 cells are plotted by blue boxes and red circles, respectively.

In order to further the analysis, we used PC classifiers and constructed a discrimination model based on LDA. The discrimination plot of LDA presented in Figure 3 shows good separation of normal and cancer cells. Results are tabulated in a confusion matrix in Table 1. Constructed model achieved 98% discrimination accuracy with 96% sensitivity and 100% specificity. 

#### 2.2.3. Support Vector Machine Analysis

Unlike LDA in which data are expected to be normally distributed, SVM makes no assumptions to the data and has gained much popularity among machine learning methods. To further test the applicability of other supervised learning model, we employed the SVM algorithm and the resultant confusion matrix is given in Table 2. Indeed, the constructed SVM model with linear classification and 10-fold cross validation could achieve superior discrimination with training accuracy of 100% and validation accuracy of 98%, as shown in Table 2.

#### 2.2.4. Multivariate Curve Resolution Analysis

**Figure 4:**
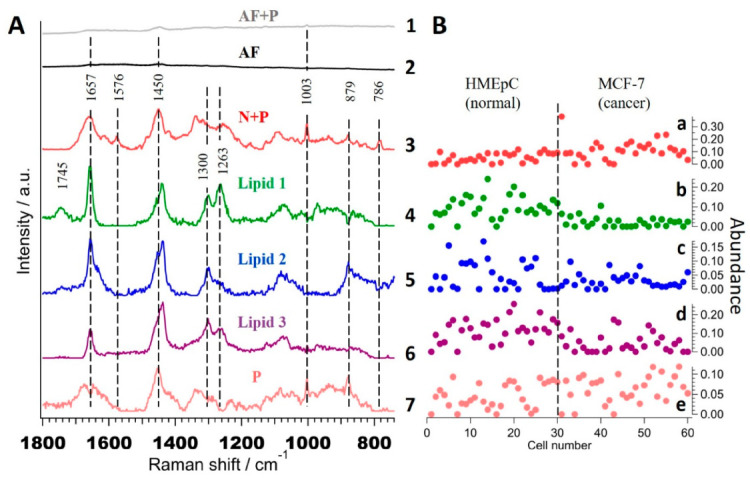
Seven components MCR-ALS analysis. (**A**)The extracted spectral components, (**1**) autofluorescence with protein [AF + P], (**2**) autofluorescence [AF], (**3**) nucleic acid with protein [N + P], (**4**) Lipid 1, (**5)** Lipid 2, (**6**) Lipid 3 and (**7**) Protein [P]. (**B**) Abundance profiles of (**a**) N + P, (**b**–**d**) lipid 1-3 and (**e**) protein, respectively. Broken line in B separates HMEpC and MCF-7 cells.

In order to understand molecular level differences and to establish a reasonable basis for successful discrimination by statistical methods such as LDA or SVM, we performed exploratory MCR-ALS analysis to obtain pure chemical components. Extracted spectral profiles of 7 components from the MCR-ALS model are presented in Figure 4A.

Unlike the results of PCA, these spectral profiles are meaningful as they correspond to pure molecular species or groups. Respective abundance profiles obtained from ‘H’ matrix of seven components MCR-ALS analysis, i.e., contribution of each component in single cells are shown in Figure 4B. Component 1 (Figure 4A(1)) with bands at 1003 cm^−1^, 1450 cm^−1^, and 1657 cm^−1^ with broad background can be assigned to that part of autofluorescence which coexists with some proteins while component 2 (Figure 4A(2)) to commonly observed autofluorescence background in Raman spectra of biological samples under this excitation conditions. Component 3 (Figure 4A(3)) containing bands typical to that of proteins at 879 cm^−1^, 1003 cm^−1^, 1657 cm^−1^ and nucleic acids at 786 cm^−1^ and 1576 cm^−1^ could be assigned to ‘nucleic acid + protein’ (denoted as ‘N + P’) that coexist together. Its abundance profile (Figure 4B(a)) suggests slightly higher concentration in MCF-7 cells. Interestingly, components 4–6, which seem spectrally similar, were separated as independent components. Bands at 1263 cm^−1^, 1300 cm^−1^, 1440 cm^−1^, and 1657 cm^−1^ indicate that these are lipids and hence named as ‘Lipid 1’ (Figure 4A(4)), ‘Lipid 2’ (Figure 4A(5)), and ‘Lipid 3’ (Figure 4A(6)). Their abundance profiles indicate ‘Lipid 1’ (Figure 4B(b)) to be lower in MCF-7 compared to HMEpC cells whereas no significant difference can be observed in other two lipids (Figure 4B(c,d)). Finally, component 7 (Figure 4A(7)) can be assigned to ‘proteins’ (denoted as ‘P’) alone, based on the spectral profile with no significant difference in their abundance.

**Figure 5:**
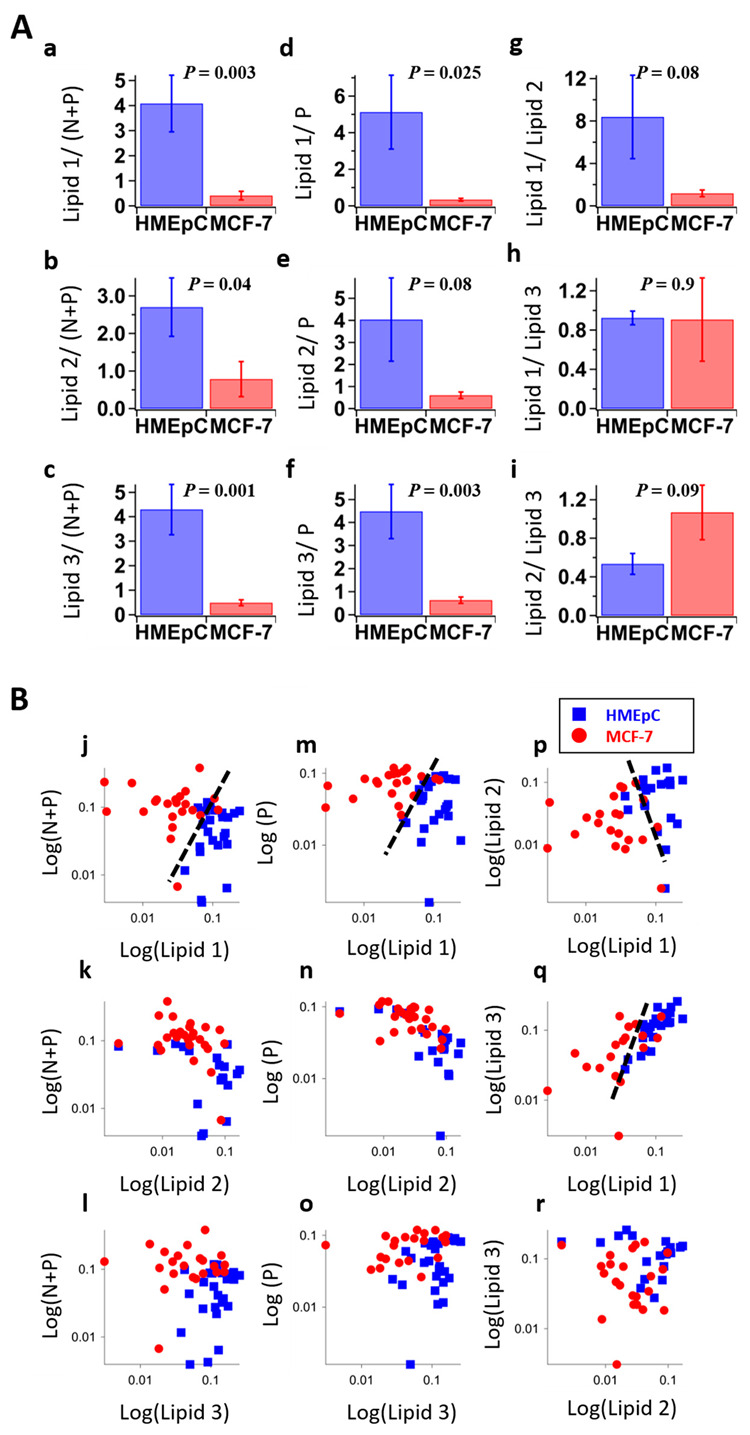
Discrimination analysis by MCR-ALS. (**A**) Relative abundance of MCR-ALS extracted components, (**a**) Lipid 1/ (N + P), (**b**) Lipid 2/ (N + P), (**c**) Lipid 3/ (N + P), (**d**) Lipid 1/P, (**e**) Lipid 2/P, (**f**) Lipid 3/P, (**g**) Lipid 1/Lipid 2, (**h**) Lipid 1/Lipid 3, (**i**) Lipid 2/Lipid 3. N + P: nucleic acid with protein, P: protein. Error bars are standard error of mean. p values obtained by t-test were denoted on top of histograms. (**B**) Scatter plots of each logarithmic abundance, (**j**) Lipid 1 vs. (N + P), (**k**) Lipid 2 vs. (N + P), (**l**) Lipid 3 vs. (N + P), (**m**) Lipid 1 vs. P, (**n**) Lipid 2 vs. P, (**o**) Lipid 3 vs. P, (**p**) Lipid 1 vs. Lipid 2, (**q**) Lipid 1 vs. Lipid 3, (**r**) Lipid 3 vs. Lipid 2. Some labels of measured cells were omitted in those plots since the values of abundance were calculated into zero by MCR-ALS. Broken lines serve as visual guides to separate two groups of cells.

Even though we get concentration information from MCR-ALS analysis, it should not be compared directly as it is not an absolute quantity. Therefore, it is safe to calculate average relative abundance of extracted components to understand meaningful trends. Figure 5A shows relative concentrations along with their standard error of three separated lipid components to ‘N + P’ (Figure 5A(a–c)), to ‘P’ (Figure 5A(d–f)) and to other lipids (Figure 5A(g–i)). Of all nine ratios, four of them; ‘Lipid 1’ to’ N + P’ or ‘P’ (Figure 5A(a–d)) and ‘Lipid 3’ to ‘N + P’ or ‘P’ (Figure 5A(c–f)) seem to have statistically significant differences. Further to perform objective discrimination based on obtained pure molecular information, we constructed scatter plots to visualize all nine combinations in a similar fashion (Figure 5B). Although several of them seem to show a fair degree of separation (as indicated by broken lines in Figure 5B(j,m,p,q)), considering statistical averages, we could conclude that ratios involving ‘Lipid 1’ to other biomacromolecules such as nucleic acids and proteins serve as reliable “Raman spectral marker” for discriminating cancer from normal cells. Moreover, it is important to note that though scatter plots show lower discrimination than some of the other chemometric methods, this disadvantage is overcome by the advantage of the physically meaningful spectra.

### 2.3. Molecular Assignment of MCR-ALS Extracted Lipid Components

**Figure 6:**
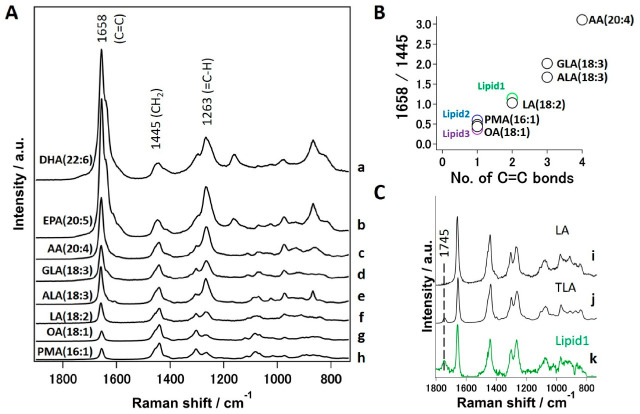
Molecular level assignment of Raman spectral marker. (**A**) Standard Raman spectra of various unsaturated fatty acids normalized using 1445 cm^−1^ band; (**a**) docosahexaenoic acid(DHA), (**b**) eicosapentaenoic acid (EPA), (**c**) arachidonic acid (AA), (**d**) γ-linolenic acid (GLA), (**e**) α-linolenic acid (ALA) (**f**) linoleic acid (LA), (**g**) oleic acid (OA) and (**h**) palmitoleic acid (PMA). (**B**) Unsaturation index plot. Relative intensity ratio of C=C/CH2 vs. number of C=C bonds in standard unsaturated fatty acids. Relative intensities obtained from three lipids on MCR-ALS components are also plotted and denoted in color. (**C**) Comparison of standard (**i**) LA, (**j**) Trilinolein (TLA) and (**k**) Lipid 1 extracted by MCR-ALS.

Now that we have identified sensitive lipid spectral markers, it is necessary to assign these components at the molecular level to develop an objective method to discriminate cancer cells from normal ones. To begin with, all three lipid components (Figure 4A(4,5,6)) show bands at 1657 cm^−1^ and 1263 cm^−1^ corresponding to -C=C- stretching and =C-H modes, respectively. Therefore, we can safely say that none of the three components are saturated lipids. In order to screen for potential candidates, we measured a series of standard fatty acids from palmitoleic acid with unsaturation index of 1 to docosahexaenoic acid with 6 double bonds to cover a wide range of polyunsaturated fatty acids (PUFA) as given in Figure 6A.

For further comparison of fatty acid standards, we normalized these spectra with band area of 1445 cm^−1^. We can observe that intensity of 1658 cm^−1^ greatly increases with an increasing number of double bonds. In fact, it is well known that Raman intensity of C=C stretching mode is directly proportional to the number of double bonds in the molecule. Therefore, it is rather straightforward to construct a calibration model to predict the unsaturation index from measured Raman spectra by calculating Raman intensity at 1658 cm^−1^ (C=C stretching vibration) to that at 1445 cm^−1^ (CH2 deformation), i.e., 1658/1445 [17]. Indeed, a linear relationship is observed when a ratio of 1658/1445 is plotted against number of double bonds in chemical structure of fatty acids as shown in Figure 6B. To predict the molecular structure of MCR-ALS extracted lipid components, we estimated the ratio of 1658/1440 in a similar fashion and compared with the constructed model as marked in Figure 6B. We could therefore assign ‘Lipid 1’ to di-unsaturated fatty acid (linoleic acid, LA) whereas ‘Lipid 2’ and ‘Lipid 3’ could be assigned to mono-unsaturated fats. It is important to note that ‘Lipid 1’ contains a Raman band at 1745 cm^−1^ corresponding to C=O stretch of esters. Since the focus is to mainly identify ‘Lipid 1’ in an unambiguous manner, we further measured trilinoleic acid (TLA), a triglyceride (TG) with three linoleic acid groups. A comparison reveals a perfect match between TLA (Figure 6C(j)) and ‘Lipid 1’ (Figure 6C(k)) as opposed to simple LA (Figure 6C(i)), in which 1745 cm^−1^ band is not observed as shown in Figure 6C. Therefore, we believe the relative content of TGs with high LA content is the main factor that helped to discriminate normal (HMEpC) and cancer (MCF-7) cells. Although ‘Lipid 2’ and ‘Lipid 3’ have been identified as mono-unsaturated fats, further unambiguous assignment to the likes of Oleic acid (18:1) or palmitoleic acid (16:1) could not be achieved in this study.

## 3. Discussion

The choice of breast cancer cell line for this work (MCF-7) was established from invasive ductal carcinoma (IDC) of a Caucasian patient and the cells are known to be estrogen (ER) and progesterone receptor (PgR)-positive. It is important to note that ER and PgR-positive IDC is the most common subtype accounting for >70% of breast cancers [19]. Therefore, analysis of such a cancer cell line adds meaningful value to understanding Raman spectral markers. Indeed, many researchers have used MA such as PCA, LDA, and SVM for RS data of cancers for a long time and reported marked differences in proteins and fat profiles in general, which corroborates well with this study [20,21,22,23,24]. Although these methods discriminated cancers well, as can also be seen from our own data, they do not give insights into the chemical changes responsible for diagnosis, thereby making it difficult to be translated to clinics. To overcome this, Haka et al. developed a method to model tissue spectra as linear combinations of known components and succeeded in discriminating cancers with some chemical information. Indeed, they showed that relatively low abundance of fats could be used as an indicator to distinguish breast cancer tissues [11,25]. However, such analysis has several assumptions and may overlook underlying pathology. Other researchers also reported decreased overall lipid content in human breast cell/biopsy samples compared to normal breast cells/tissues using RS but without molecular level information [15,16,26,27,28]. Our results specifically showed that relative abundance of linoleate-rich glyceride to other biomacromolecules, such as nucleic acids and proteins, to be the major difference and possibly the reason for successful discrimination of breast cancer cells from normal epithelial cells. Interestingly, a previous attempt by Sixian et al. could not find strong correlation with PUFA and protein by Raman spectroscopy [29]. We believe it was because they calculated the ratio considering all fats as a single entity. It is important to note from this study that, although there are three groups of unsaturated fatty acids, only a linoleate-rich component could serve a reliable discrimination index.

Alterations in lipid metabolism have been shown to play a critical role in development, promotion, and maintenance of cancers [30,31]. Therefore, reprogramming of lipid metabolism is being considered a hallmark of malignancy and can be used as a novel target for anti-cancer strategy [32,33]. In particular, the role of unsaturated fatty acids including LA is of great importance as it is used for synthesizing arachidonic acid (AA). For example, cyclooxygenase (COX) enzymes convert AA to bioactive lipids such as prostaglandins (PG), which play key roles in adhesive, migratory, and invasive behavior of cells during development and progression of breast and other cancers [34,35,36]. Therefore, we suspect from our results that a certain amount of AA could have been used up for the synthesis of PG, thereby depleting LA-rich TG in MCF-7 cells.

From the nature of the analysis used in this study, one might expect that several protein and/or saccharide components should also have been extracted. However, it is important to understand the limitations involved. Since we use spontaneous Raman microspectroscopy, one of the main limitations in detecting several more biomolecular components is their local intracellular concentrations. Limitation to resolve multiple components arises from the inherent nature of MCR-ALS with applied penalties (L1- and L2-norms). It is not possible to unmix two spectral components if there is no difference in their intracellular distribution pattern. Essentially, such components are treated as a single component. Considering the above limitations, it is understandable as to why weak or minor molecular components such as saccharides could not be detected/separated in this study.

## 4. Materials and Methods

### 4.1. Cell Culture

MCF-7 malignant breast cancer cell line was cultured in DMEM low glucose without phenol red (Thermo Fisher Scientific, Tokyo, Japan) with added supplements (0.1 mM sodium pyruvate, 2 mM L-Glutamine, 1% (*v*/*v*) antibiotics and 5% (*v*/*v*) fetal bovine serum). HMEpC primary cells obtained from normal mammary glands (Cell Applications, Inc., San Diego, CA, USA) as control were cultured in Human Mammary Epithelial Cell Media (TOYOBO Life Science, Osaka, Japan). Both MCF-7 and HMEpC cells were incubated at 37 °C and 5% CO_2_. Cells were sub-cultured at ~80% of cell confluence and Raman spectra were obtained from cells incubated for 3 days after gently washing with PBS (-) on Poly-L-Lysine-coated glass bottom dish.

### 4.2. Raman Microspectroscopy

Raman spectra were measured using a homemade confocal Raman micro-spectrometer [37,38]. An excitation part consists of He-Ne laser (632.8 nm) coupled to an inverted microscope (IX70, Olympus) with an oil immersion objective lens (100×, NA = 1.3) to focus the excitation laser on specific points of cultured cells. Stokes Raman scattered light was collected using the same objective lens in back scattering geometry using a long pass filter. To improve axial resolution, a confocal pinhole of 50 μm was used in collection path. A polychromator (Chromex, 250IS) dispersed the scattered light and was detected with a CCD device (Princeton instruments, Spec-10) cooled at −120 °C with liquid nitrogen. To achieve optimal throughput while measuring the whole finger print region, we used a 600 g/mm grating and set the slit width of polychromator to 50 μm. All Raman measurements were done at room temperature (22 °C) and the laser power was set to 4 mW at the sample position. Raman spectra were obtained from 5 random points in each cell with an exposure of 30 s/point. A total of 60 cells (30 cells for each kind) were measured and averaged. For lipid standards, several unsaturated fatty acids including docosahexaenoic acid (DHA), eicosapentaenoic acid (EPA), arachidonic acid (AA), γ-linolenic acid (GLA), α-linolenic acid (ALA), linoleic acid (LA), oleic acid (OA), and palmitoleic acid (PMA) purchased from Tokyo Chemical Industry Co., Ltd, Tokyo, Japan. were measured under the same conditions.

### 4.3. Data Analysis

Data pre-processing such as dark light subtraction, cosmic ray removal, and data de-noising by SVD were performed by IGOR Pro (Wavemetrics, Portland, OR, USA). Generally, no Raman bands are expected in the so-called silent region between ~2800 cm^−1^–1800 cm^−1^. Therefore, a preliminary analysis of Raman spectra in the whole fingerprint region between ~1800 cm^−1^–370 cm^−1^ was carried out. Since no significant Raman band was observed except for strong contribution from background (shown in Supplementary Materials Figure S1), the fingerprint region between 739 ~ 1800 cm^−1^ was chosen for multivariate analysis.

#### 4.3.1. Discriminant Analysis

The first PCA was performed on mean-centered data using NIPALS algorithm with random cross validation to extract principal components (PC). Using prior knowledge of principal components, an LDA model for two classes was constructed by including the first 4 PC scores assuming equal prior possibilities. Furthermore, to construct an SVM model, nu-SVM with linear kernel type was employed with 10-fold cross validation. PCA, LDA and SVM were performed using Unscrambler (Camo Analytics, Oslo, Norway).

#### 4.3.2. MCR-ALS

In MCR-ALS, a low-rank approximation of matrix A is obtained by solving the following Equation (1):

A = WH(1)

in which A is an m x *n* non-negative Raman hyperspectral data matrix. All elements of W (m × k matrix) and H (k × *n* matrix), which represent spectral components and corresponding abundances respectively, are restricted to be non-negative. Parameter k represents the number of spectral components and was set to 7 in this study based on SVD analysis [39]. First, 7 SVD components were used as initial points for further analysis. W and H were iteratively calculated to refine the quality of approximation using alternating least squares so that the Frobenius norm ||A-WH||2 was minimized with non-negative constraints W ≥ 0 and H ≥ 0. To obtain sparser solutions, additional L1 penalty term (lasso regression) of α2 = 0.005 was applied as

(WTW + α2E)H = WTA(2)

where E is a k × k matrix whose elements are all unity. Additionally, L2 penalty term (ridge regression) of β2 = 0.005 was also applied as follows:

(HHT + β2I)W = HAT(3)

where I is a k × k identity matrix. MCR-ALS was performed using a homemade program specifically developed for Raman spectroscopic applications using Python [39].

## 5. Conclusions

In this study, we tried to address the age-old problem of efficiently extracting hidden information from chemically rich Raman hyperspectral data. In addition to demonstrating the utility of discrimination analysis such as LDA and SVM, we developed and employed MCR-ALS with non-negative constraints to extract physically meaningful Raman spectra using mammary epithelial cells and breast cancer cells as a model case. In a truly exploratory fashion, without a priori information, we obtained various biomolecular spectra including three individual lipid groups and successfully identified relative ratios of linoleate-rich glyceride as the Raman spectral marker and molecular basis for objective diagnosis of breast cancer. We would like to emphasize that this is the first report that discusses cancer pathology in detail while discriminating breast cancer cells unambiguously using specific fatty acid content in chemometrics-assisted RS. However, further studies are necessary to determine whether the differences in linoleate-rich triglycerides can be directly related to cancer states. Although both cell lines used in this study are of epithelial source, it is important to understand that most tumors are like organs and have more than one type of cell. Therefore, while the model holds true to this breast cancer cell line with 633 nm excitation, it is imperative that we further test on large numbers of other cell lines and with different excitations wavelengths as well to have general consensus. Once established, spectral markers identified in the present study being at the cellular level have the potential to be used as an adjunct or even an alternative to cytological diagnosis, especially because specimens for cytology have scattered cells in them that are appropriate for RS. Moreover, RS can be performed on any biological sample including cells, tissues and body fluids etc. We believe such an approach when further developed can be adopted to real clinical applications for rapid yet objective diagnosis of certain types of cancers.

## Figures and Tables

**Table 1:** Confusion matrix of linear discriminant analysis (LDA) Actual.

		HMEpC	MCF-7
**Predicted**	HMEpC	30	1
	MCF-7	0	29

**Table 2:** Confusion matrix of SVM.

Actual			
		HMEpC	MCF-7
**Predicted**	HMEpC	30	0
	MCF-7	0	30

## Data Availability

Data is contained within the article or Supplementary Material.

## Supplementary Materials

Supplementary materials can be found at https://www.mdpi.com/1422-0067/22/2/800/s1. Figure S1: Preliminary MCR analysis of whole fingerprint region (1800–370 cm^−1^).

## Abbreviations

AA	Arachidonic acid
ALA	α-linolenic acid
DHA	Docosahexaenoic acid
EPA	Eicosapentaenoic acid
GLA	γ-linolenic acid
HMEpC	Human mammary epithelial cells
LA	Linoleic acid
LDA	Linear discriminant analysis
MA	Multivariate analysis
MCF-7	Michigan cancer foundation-7
MCR-ALS	Multivariate curve resolution-alternating least squares
OA	Oleic acid
PC	Principal components
PCA	Principal components analysis
PMA	Palmitoleic acid
PUFA	Polyunsaturated fatty acids
RS	Raman Spectroscopy
SVD	Singular value decomposition
SVM	Support vector machine
TLA	Trilinoleic acid
