# Supplementary material for: Identification of Molecular Basis for Objective Discrimination of Breast Cancer Cells (MCF-7) from Normal Human Mammary Epithelial Cells by Raman Microspectroscopy and Multivariate Curve Resolution Analysis
Source: Int J Mol Sci. 2021 Jan 14;22(2):800. doi: 10.3390/ijms22020800 (PMC7830327; doi:10.3390/ijms22020800)
Supplement: Supplementary file 1 [file ijms-22-00800-s001.pdf]

International Journal of *Molecular Sciences*

Article

Identification of Molecular Basis for Objective Discrimination of Breast Cancer Cells (MCF-7) from Normal Human Mammary Epithelial Cells by Raman Microspectroscopy and Multivariate Curve Resolution Analysis

Keita Iwasaki [1], Asuka Araki [2], Murali Krishna C [3], Riruke Maruyama [2], Tatsuyuki Yamamoto [4,5,*] and Hemanth Noothalapati [5,6,*]

1 The United Graduate School of Agricultural Sciences, Tottori University, Tottori 680-8550, Japan; d17a3003@matsu.shimane-u.ac.jp
2 Department of Organ Pathology, Faculty of Medicine, Shimane University, Izumo 693-8501, Japan; asuka@med.shimane-u.ac.jp (A.A.); hm5995@med.shimane-u.ac.jp (R.M.)
3 Advanced Centre for Treatment, Research and Education in Cancer, Tata Memorial Centre, 410-210 Navi Mumbai, India; pittu1043@gmail.com
4 Faculty of Life and Environmental Science, Shimane University, Matsue 690-8504, Japan
5 Raman Project Center for Medical and Biological Applications, Shimane University, Matsue 690-8504, Japan
6 Research Administration Office, Shimane University, Matsue 690-8504, Japan
* Correspondence: tyamamot@life.shimane-u.ac.jp (T.Y.); nvhnag@life.shimane-u.ac.jp (H.N.)

Supplementary Information

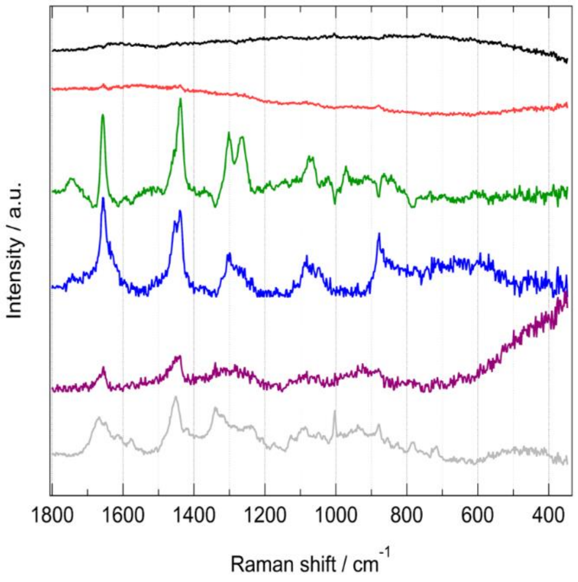

Figure S1. Preliminary MCR analysis of whole fingerprint region (1800–370 cm^{-1}). No significant bands were observed below 750 cm^{-1}.
